# Estimating the response and economic burden of rheumatoid arthritis patients treated with biologic disease-modifying antirheumatic drugs in Taiwan using the National Health Insurance Research Database (NHIRD)

**DOI:** 10.1371/journal.pone.0193489

**Published:** 2018-04-06

**Authors:** Qiang Shi, Ko-Jen Li, Tamas Treuer, Bruce C. M. Wang, Carol L. Gaich, Chien-Hsun Lee, Wen-Shuo Wu, Wesley Furnback, Chao-Hsiun Tang

**Affiliations:** 1 Eli Lilly & Co., Shanghai, China; 2 National Taiwan University, Taipei, Taiwan; 3 Eli Lilly & Co., Budapest, Hungary; 4 Elysia Group Ltd., Taipei, Taiwan; 5 Eli Lilly & Co., Indianapolis, Indiana, United States of America; 6 Eli Lilly & Co., Taipei, Taiwan; 7 Taipei Medical University, Taipei, Taiwan; VU University Medical Center, NETHERLANDS

## Abstract

**Background:**

Previous studies in Taiwan utilizing the Taiwan’s National Health Insurance Database (NHIRD) have estimated the direct healthcare costs of RA patients, but they have not focused on patients on bDMARDs, or considered patients’ response to therapy.

**Objectives:**

The objective of this study was to estimate the rate of inadequate response for patients newly treated with biologic disease-modifying antirheumatic drugs (bDMARDs) as well as their costs and resource use.

**Methods:**

Data were from the catastrophic illness file within the NHIRD from 1/1/2009 to 12/31/2013. Patients with RA, which was categorized by the presence of a catastrophic illness card, that were previously bDMARD-naïve, were included in this study if they initiated their first bDMARD during the index period. The index period included all of 2010, a pre-index period consisting of the index date– 365 days, and a follow-up period including the index date to 365 days post-index, were also included. Previously biologically-naïve patients were indexed into the study on the date of their first claim for a bDMARD. A validated algorithm was used to examine the rate of inadequate response (IR) in the biologically-naïve cohort of patients. Inadequate responders met one or more of the following criteria during their year of follow-up: low adherence (proportion of days covered <0.80); switched to or added a second bDMARD; added a new conventional synthetic DMARD (csDMARD); received ≥1 glucocorticoid injection; or increased oral glucocorticoid dosing. All-cause mean annual direct costs and resource use were measured in the year of follow-up. Costs were converted from NT$ to USD using 1 NT$ = 0.033 USD.

**Results:**

A total of 818 patients with RA initiated their first bDMARD (54% etanercept and 46% adalimumab) in 2010. After one year of follow-up, 32% (n = 258) were classified as stable, 66% (n = 540) had an IR, and 2% (n = 20) were lost to follow-up. During the follow-up period mean annual total direct costs were $16,136 for stable patients compared to $14,154 for patients with IR. Mean annual non-medication direct costs were $937 for stable patients and $1,574 for patients with IR. Mean annual hospitalizations were higher for patients with IR (0.46) compared to stable patients (0.10) during the one year follow-up period.

**Conclusions:**

The majority of patients that were previously naïve to bDMARDs had an IR to their first bDMARD during the year of follow-up. Patients with an IR had numerically increased all-cause resource utilization and non-medication costs during the follow-up period compared to patients with stable disease. This level of IR suggests an unmet need in the RA treatment paradigm.

## Introduction

Rheumatoid arthritis (RA) is a chronic, systemic inflammatory disease characterized by synovial inflammation, swelling, cartilage and bone destruction, and other systemic features [1]. Within low-to-middle income countries, the prevalence of RA is estimated to be slightly lower for Southeast Asian countries than those in Europe, the Americas, and Western Pacific regions [2]. The mean age-adjusted incidence of RA in Taiwan is estimated to be 15.8 per 100,000 [3,4]. The estimated prevalence of RA in Taiwan was 99.6 per 100,000 in 2007 [4].

Patients with RA often experience morning stiffness, pain and limitations of motion, and general difficulty performing daily activities [5]. Extra-articular and systemic manifestations are commonly associated with RA patients and affect various tissues and organ systems [6,7]. These extra-articular symptoms include fever, fatigue, malaise, depression, weakness, and/or cardiovascular disease (CVD) [8,9].

Patients with RA can experience comorbidities such as depression, asthma, cardiovascular events (myocardial infarction, stroke), solid cancers, and chronic obstructive pulmonary disease. However, there is high inter-country variability in the prevalence of comorbidities [10]. RA comorbidities have been shown to shorten patient life expectancy [11,12], primarily due to the increased prevalence of CVD, a greater incidence of infections, and cancer [13–17]. Furthermore, osteoporotic fractures are more commonly observed in RA patients and can lead to functional decline [18,19]. A retrospective database analysis of Taiwan’s National Health Insurance Research Database (NHIRD) using data from 2004 to 2010 found a substantially increased incidence rate of hip fractures in younger patients with RA vs. those without across all age groups [20].

There are three main medication classes used to treat RA, depending on the severity of disease and symptoms. These classes include nonsteroidal anti-inflammatory drugs (NSAIDs), corticosteroids, and disease-modifying antirheumatic drugs (DMARDs) [21]. Furthermore, the DMARD class of medications include conventional synthetic DMARDs (csDMARDs), targeted synthetic DMARDs (tsDMARDs), and biologic DMARDs (bDMARDs) [22]. csDMARDs include medications such as methotrexate and leflunomide, while tsDMARDs include Janus Kinase (JAK) inhibitors such as tofactinib. bDMARDs include several different medications using different mechanisms of action including tumor necrosis factor (TNF)-inhibitors, T-cell inhibitors, interleukin (IL)-1 receptor antagonists, and anti-IL-6.

For patients utilizing biologics, a significant proportion either do not respond, discontinue, or lose efficacy over time [23–25]. Response to therapy can vary for patients based on disease factors and treatment choice. Typically biologic therapy is initiated using TNF-inhibitors. However, an estimated 30% of patients have an inadequate response to them [23]. While patients will often switch to a different TNF-inhibitor rather than a different class of medication, research has shown inadequate response to an initial TNF-inhibitor to be a predictor of response and tolerance of a second TNF-inhibitor [26].

In Taiwan, patients with RA must fit the following treatment criteria: 1) fulfill ACR criteria for RA, 2) have continuously active RA, which is defined as a disease activity score (DAS) 28 score of >5.1, and 3) have failed csDMARD treatment, defined as being treated with at least two csDMARDs which resulted in no significant efficacy in order to be considered for reimbursement of a bDMARD [27]. In Taiwan, reimbursement for the continuous use of biologics is limited to three years before patients must discontinue with a mandatory break before re-applying for reimbursement [27]. Rather than limiting time on therapy, guidelines have increasingly called for a treat-to-target strategy [28]. This treatment strategy involves continuous assessment of disease activity every 1–3 months, with adjustments made to therapy if patients are not reaching either remission or low disease activity.

This study attempts to quantify the number of patients not responding to their newly initiated bDMARD therapy, their healthcare resource utilization (HCRU) and the economic burden in Taiwan, using the NHIRD. Previous studies in Taiwan utilizing the NHIRD have estimated the direct healthcare costs of RA patients, but they have not focused on patients on bDMARDs, or considered patients’ response to therapy [29–31]. This study will provide evidence for decision makers to understand the real-world rate of response to therapy, and the differences in HCRU and costs between patients responding, and not responding to therapy.

## Methodology

### Data source

This study utilized Taiwan’s National Health Insurance Database, which is a population-based claims database covering over 99% of Taiwan’s population. Claims data from 1/1/2009 to 12/31/2013 were included in the study. Subjects included in the catastrophic illness file, which is a registry file containing all cases of patients with major diseases, were analyzed for their RA diagnosis.

### Study design

**Fig 1:**
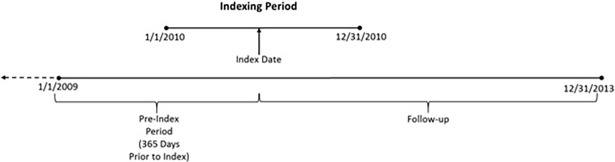
Study timeline.

This retrospective, longitudinal study utilized data from 1/1/2009 to 12/31/2013 (Fig 1). The index period ran from 1/1/2010 to 12/31/2010, with the pre-index period defined as the 12-months prior to index.

Previously bDMARD-naïve RA patients initiating a bDMARD during the index period were included in the study if they had any healthcare event with an International Classification of Diseases, Ninth Revision, Clinical Modification (ICD-9-CM) code 714.xx (excluding 714.3), a claim for a bDMARD in the index period, and the presence of a catastrophic illness card for RA. Patients were excluded from the study if they: 1) were aged <18 years at index; 2) were not in the database in the pre-index period (ignoring breaks of <30 days); 3) had ≥1 claim for any of the following conditions: comorbid psoriasis or psoriatic arthritis (696.xx), ankylosing spondylitis (720.0x), Crohn’s disease (555.xx), ulcerative colitis (556.xx) and juvenile chronic polyarthritis (714.3x), at any time during the study period; or 4) had a diagnosis of any solid tumors or hematologic malignancy (140-208.xx), except for non-melanoma skin cancer (173.xx) or human immunodeficiency virus (HIV)/acquired immune deficiency syndrome (AIDS) (042.xx, 043.xx, 044.xx) at any time during the study period.

### Study population

Patients identified as bDMARD-naïve were followed for one year post-index to evaluate their response. Patients were divided into three possible cohorts: 1) bDMARD stable disease; 2) bDMARD-Inadequate Response (IR); or 3) bDMARD Unknown status. Response was measured using an algorithm adapted from Curtis et al. to Taiwan [32]. The algorithm considered five criteria for response. Patients meeting all five criteria were considered to have stable disease. Patients meeting one or more of the IR criteria were considered inadequate responders, and those lost to follow-up were considered to have unknown status. The response criteria included: (1) adherence (<80% proportion of days covered); (2) a switch or add of a bDMARD; (3) the addition of a new non-biologic DMARD; (4) more than one glucocorticoid joint injection; and (5) an increase in the dose of an oral glucocorticoid. The algorithm also included the criteria of an increased dose in bDMARD to be a measure of inadequate response. In localizing this algorithm to Taiwan we did not consider this criteria as patients are not able to increase their dose per the reimbursement guidelines [27].

### Statistical analysis

Baseline demographics (age, gender, comorbidities) and treatment mix for each of the cohorts were measured. Comorbidities were measured using a claims-based algorithm to estimate patients’ Charlson Comorbidity Index (CCI) [33]. A total of three years of follow-up post-index were analyzed to evaluate switching patterns, HCRU, and costs. Included bDMARDs were abatacept, adalimumab, etanercept, golimumab, and tocilizumab. The tsDMARD tofactinib was not reimbursed during the study period and was therefore not included in the analyses. Direct costs were expressed as mean annual costs for each of the cohorts, and separated into major categories including: outpatient, inpatient, emergency department (ED), and medication. Resource utilization was the mean annual quantity of service (hospital admissions, days of hospitalization, outpatient visits, and ED visits) used. All outcomes were measured for each of the patient’s groups. All analyses were for descriptive purposes only and no formal statistical inference testing were conducted. The software SAS (version 9.4) was used in management and descriptive analysis of data. All costs were converted to 2017 USD using an exchange rate of 1 TWD = 0.033 USD [34].

## Results

### Incidence of RA patients initiating a biologic

**Fig 2:**
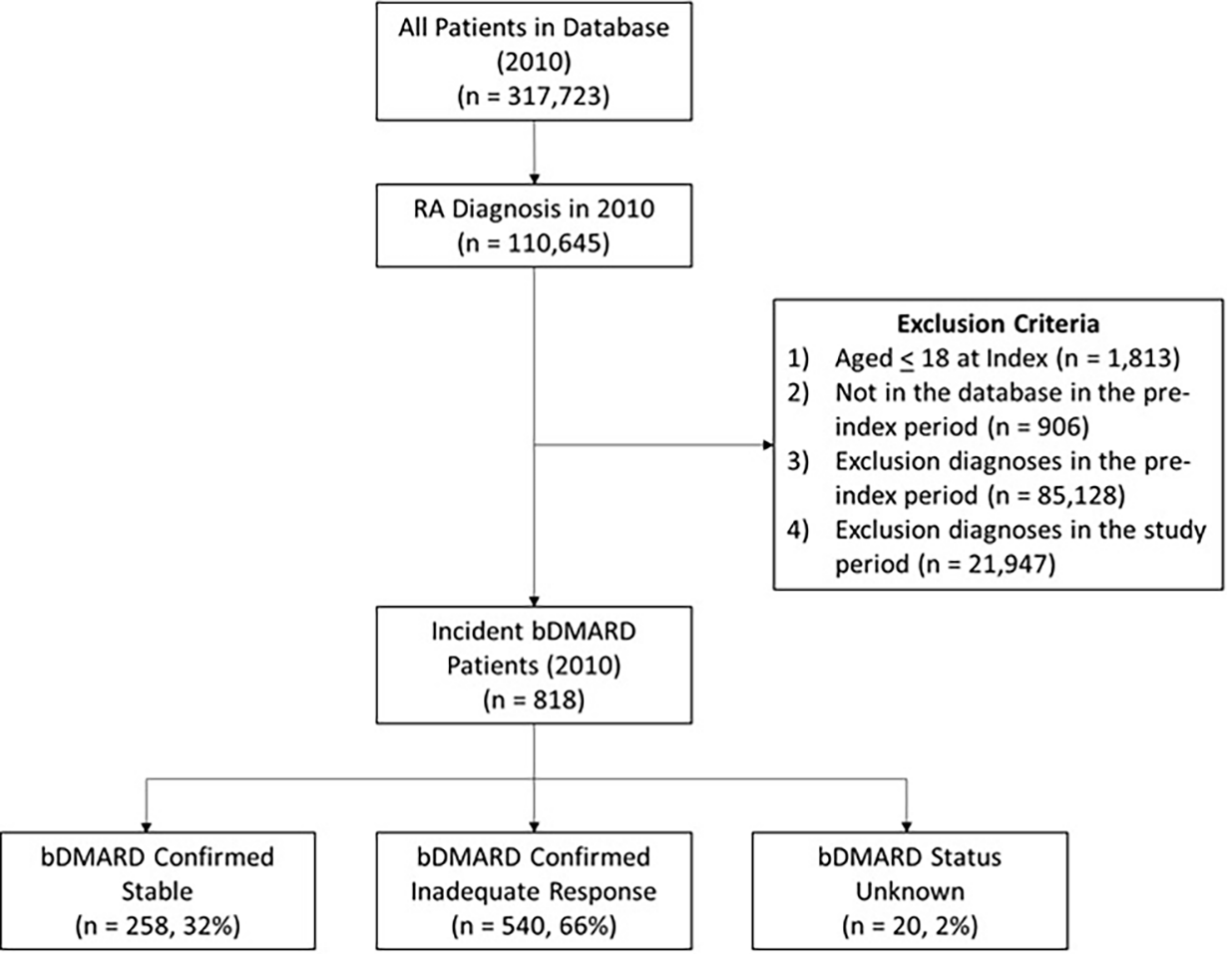
Selection of study subjects.

A total of 110,645 patients with a diagnosis of RA in 2010 were included in the study. Of these patients, a total of 818 fit the study criteria to be classified as patients initiating a bDMARD in 2010 (Fig 2).

Patients initiating a bDMARD in 2010 had a mean age of 55.5 years, were 84% female, and had a mean CCI of 1.65 (Table 1). All patients either initiated adalimumab (n = 375, 46%) or etanercept (n = 443, 54%) as their first biologic therapy.

**Table 1:** Baseline characteristics of bDMARD naïve patients by cohort.

Description	All incident bDMARD Patients	Confirmed Stable	Inadequate Responders	Status Unknown
Number	818	258	540	20
Mean Age (SD)	55.5 (13.1)	54.1 (11.6)	55.7 (13.4)	69.1 (13.9)
Gender				
Male, n (%)	133 (16%)	44 (17%)	83 (15%)	6 (30%)
Female, n (%)	685 (84%)	214 (83%)	457 (85%)	14 (70%)
Charlson Comorbidity Index, mean (SD)	1.65 (0.98)	1.48 (0.76)	1.68 (1.02)	2.75 (1.55)

Within the bDMARD naïve cases (n = 818), after one year of follow-up, a total of 258 (32%) patients were confirmed stable, 540 (66%) patients were classified as inadequate responders, and 20 (2%) patients had the status of unknown. As shown in Table 1, patients with an inadequate response to their biologic had a higher numerically mean age than those confirmed stable (55.7 vs. 54.1 years).

Inadequate response was driven by low adherence (36%) followed by an increase in glucocorticoid dose (20%), ≥1 glucocorticoid joint injection (17%), addition of a new csDMARD (8%), and finally, switching or adding a biologic (3%).

### Medication switching

Of the 818 patients initiating a biologic in 2010, 135 (17%) switched to a second biologic during the three years of follow-up. Patients who initiated adalimumab (n = 375) switched to a second-line biologic 21% (n = 79) of the time, while those who initiated etanercept (n = 443) switched 13% (n = 56) of the time. Of the 79 patients that switched from their adalimumab first-line biologic to another, 32 (41%) switched to etanercept, 31 (39%) to tocilizumab, 9 (11%) to golimumab, and 7 (9%) to abatacept. Of the 56 patients starting on etanercept and switching, 30 (54%) switched to adalimumab, 13 (23%) to tocilizumab, 8 (14%) to golimumab, and 5 (9%) to abatacept.

**Fig 3:**
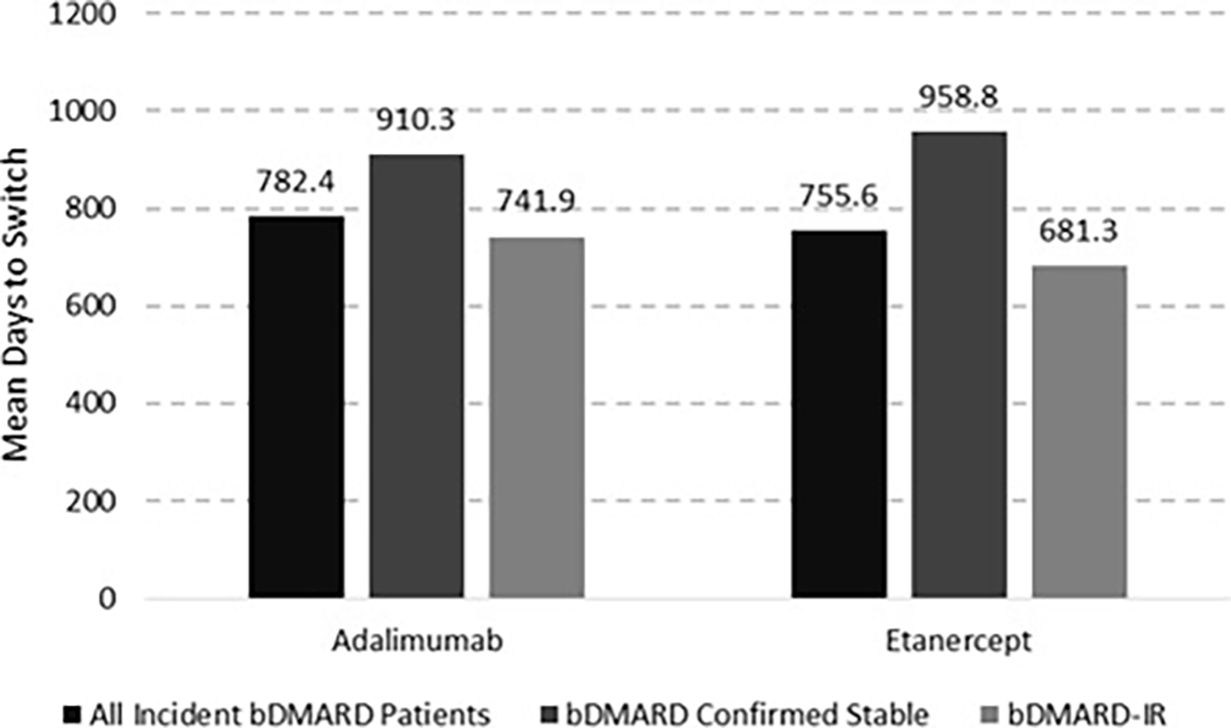
Time to switch from first-line to second-line treatment.

Of the patients who switched to another biologic medication, the mean time to switch from first-line therapy for all bDMARD naïve patients was 782.4 days for patients that started on adalimumab and 755.6 days for those starting on etanercept. As expected, patients with an inadequate response had a shorter time to switch to a new bDMARD than the confirmed stable cohort due to the IR algorithm. (Fig 3). The difference in mean days to switch between the stable and IR cohorts was significant for etanercept users (p = 0.0031), but not for adalimumab users (p = 0.0566).

### Healthcare resource utilization

Mean annual resource utilization was collected for each of the cohorts for years one through three (Table 2). For years one through three, the bDMARD-IR cohort of patients had higher numerically mean annual hospital admissions, hospital days, and outpatient visits.

**Table 2:** Mean annual resource utilization.

Resource Use Per Patient-Year	All Incident bDMARD Patients						bDMARD Confirmed Stable						bDMARD-IR					
	Year 1		Year 2		Year 3		Year 1		Year 2		Year 3		Year 1		Year 2		Year 3	
	Mean	SD		Mean	SD		Mean	SD	Mean	SD		Mean	SD		Mean	SD	Mean	SD		Mean	SD		Mean	SD
Number of Patients	798		785		741		258		256		243		540		529		498	
Hospital Admissions	0.34	0.74	0.48	1.35	0.55	1.24	0.10	0.34	0.27	0.78	0.39	0.97	0.46	0.85	0.58	1.54	0.63	1.34
Hospital Days	2.46	7.24	2.35	7.99	2.89	11.77	0.49	2.00	1.54	6.06	1.95	5.93	3.40	8.54	2.74	8.75	3.36	13.73
ED Visits	0.44	0.96	0.40	1.06	0.45	1.08	0.30	0.77	0.45	1.41	0.42	1.33	0.51	1.03	0.37	0.84	0.46	0.94
Outpatient Visits	40.94	21.49	39.03	20.69	40.05	22.47	37.66	20.54	37.10	19.65	37.79	20.68	42.50	21.77	39.96	21.13	41.15	23.23

### Direct costs

Direct medical costs were measured for each of the cohorts, over three years, and categorized into non-medication costs (inpatient, outpatient, and emergency department costs) and medication costs (Table 3). All bDMARD naïve patients had mean total direct costs of $14,795, $11,460, and $10,395 from years one through three, respectively. For each of these years, medication costs were the primary driver contributing 91%, 88%, and 87% of costs in years one through three, respectively.

**Table 3:** Mean annual direct costs.

Direct Costs (2017 USD)	All Incident bDMARD Patients						bDMARD Confirmed Stable						bDMARD-IR					
	Year 1		Year 2		Year 3		Year 1		Year 2		Year 3		Year 1		Year 2		Year 3	
	Mean	SD	Mean	SD	Mean	SD	Mean	SD	Mean	SD	Mean	SD	Mean	SD	Mean	SD	Mean	SD
Number of Patients	798		785		741		258		256		243		540		529		498	
Inpatient Costs	$485	$1,407	$452	$1,561	$482	$1,391	$104	$455	$307	$1,027	$394	$1,090	$667	$1,651	$522	$1,759	$526	$1,516
Outpatient Costs	$840	$926	$849	$1,066	$867	$994	$803	$1,371	$837	$1,392	$834	$1,418	$857	$610	$855	$867	$884	$700
ED Costs	$44	$127	$34	$116	$51	$135	$30	$116	$32	$124	$38	$117	$50	$131	$35	$112	$57	$142
Total Medication Costs	$13,427	$3,546	$10,126	$4,701	$8,995	$4,736	$15,198	$1,464	$11,908	$3,486	$10,467	$3,987	$12,580	$3,918	$9,263	$4,966	$8,276	$4,906
Total Costs	$14,795	$3,866	$11,460	$5,075	$10,395	$5,072	$16,136	$2,214	$13,083	$3,985	$11,734	$4,536	$14,154	$4,299	$10,674	$5,355	$9,742	$5,194

ED = Emergency Department

When responder status was considered, confirmed stable patients in general had 14% ($16,136 vs. $14,154), 23% ($13,083 vs. $10,674), and 20% ($11,734 vs. $9,742) numerically higher mean costs than the IR patient cohort over the three years post initiation, respectively. This increase in costs was driven by medication costs, which remained high for stable patients and decreased significantly for IR patients, as the primary driver of IR was low adherence. When only non-medication costs were considered, stable patients had lower mean outpatient, inpatient, and ED costs for years one through three, post initiation. In total, mean non-medication costs were 40%, 17%, and 14% numerically lower for stable patients compared to IR patients, in years one through three, respectively.

## Discussion

In this population-based analysis, a total of 3,399 naïve RA patients with a catastrophic illness card initiated either a bDMARD or csDMARD in 2010. Of these patients, 2,581 (76%) started on csDMARDs and 818 (24%) started on bDMARDs. While bDMARDs are a subsequent line of therapy, our results suggest over two-thirds of patients initiating bDMARDs are inadequate responders after one year of therapy.

Our study results are in-line with previous analyses conducted using the same algorithm in different databases. The IR rate of 66% in our study was slightly lower than the original validation of the algorithm in the Veteran Health Administration’s RA Registry of 72% [32]. The algorithm was also utilized using Medicare data from 2006–2010 for biologic-naïve patients with RA. At one year the rate of IR was 75% across all treatments (etanercept = 28%; abatacept = 26%; adalimumab = 24%; infliximab = 23%). When only considering adalimumab and etanercept, as in our study, the IR rate was 74% [32]. The lower rate of IR in our study can mostly be explained by the lack of dose increases in Taiwan, which is regulated through reimbursement guidelines. Additionally, our study only included etanercept and adalimumab as first-line therapies. The high concentration of patients on etanercept, which is a human soluble dimeric TNF receptor fusion protein, and lack of patients on infliximab, a mouse-human chimeric mAb against TNF could have contributed to patients remaining on their biologic for longer periods and decreasing the number of IR patients. Etanercept has been shown to have fewer patients develop anti-drug antibodies compared to adalimumab and infliximab [35]. The results of our study also reaffirm the 30% rate of inadequate response to initial TNF-inhibitors presented by Furst and Emery [23].

In the first year of initiation, patients with an inadequate response to bDMARDs had mean annual total direct healthcare expenditures of $14,154 compared to $16,136 for patients classified as stable. The higher costs in the stable cohort were driven by medication costs, as IR patients often discontinued their medication, reducing costs. When only non-medication costs were considered, All patients either initiated adalimumab (n = 375, 46%) or etanercept (n = 443, 54%) as their first biologic therapy. Resource utilization for IR patients was numerically higher than stable patients over the first year after initiation of therapy. This numerical increase in HCRU was 7-fold for hospital days (3.4 vs. 0.49), 4.6 times for hospital admissions (0.46 vs. 0.1), and over 1.7 times for ED visits (0.51 vs. 0.3).

A previous analysis of RA patients utilizing etanercept in 2005 in Taiwan found an annual total healthcare expenditure of NT$771,140 [36]. Patients in our study initiating etanercept had mean annual total direct costs of $14,695 (NT$445,314) in the first year after initiation. The difference between studies is driven by non-medication costs. Medication costs were similar, NT$383,670 in Hsieh et al. vs. $13,363 (NT$404,950) in our analysis. Non-medication costs were $1,332 (NT$40,364) in our study vs. NT$387,470. We believe the difference in non-medication costs are due to the timing and sample differences between studies. The Hsieh et al. study utilized data from 2005, and only included 78 etanercept users out of a total of 15,697 patients with RA. During this time period only the most severe RA patients utilized etanercept, this also contributed to the small sample size of 78 vs. 431 for our study.

The results of this study highlight an unmet need within the treatment paradigm. In Taiwan, bDMARDs accounted for nearly 57.9% ($50M USD of $86.4M USD) of direct medical costs for RA patients in 2011 [31]. Because of the high medication costs associated with biologics, the early identification of inadequate responders can help payers to redirect these costs to treatments with better health outcomes.

Future extensions of this research could include an analysis of the response to second line therapies to better understand the impact of TNF-cycling versus utilizing a treatment with a new mechanism of action for patients with inadequate response to their first therapy. Additionally, an analysis of the societal costs associated with patients considered stable vs. those with inadequate response could be explored. The results of this study could aid in the development of cost-effectiveness and budget impact models, which could help payers to understand the value and financial impact of initiating more effective medications earlier in the treatment paradigm to reduce the number of inadequate responders.

Our study includes several limitations. First, data for this study was only available through 2013. Our cohort of patients were only initiated on either adalimumab or etanercept, both TNFs, and did not examine bDMARDs with alternative mechanisms of action. Second, this treatment algorithm to determine response was developed, and preliminarily validated in the Veterans Health Administration, which is a claims–based database within the Department of Veteran Affairs (VA) RA Registry in the United States. The applicability of the algorithm outside the US, specifically Taiwan, has not previously been examined. The algorithm also relies on a set of criteria that are related to effectiveness, but not directly tied to response. Patients initiating or escalating oral glucocorticoids were said to have inadequate response, however patients could have initiated or escalated due to a comorbidity such as COPD and not due to RA. Lastly, the dataset is a claims-based dataset, which served as a proxy for clinical outcomes. The true clinical response of patients remains unknown, while the predictive algorithm has been shown to estimate the percentage of inadequate responders fairly accurately, it may be prone to misclassification. Additionally, the claims-based dataset does not include clinical variables, which limited the variables used for inclusion/exclusion in the study and could have led to bias in the study results.

## Conclusion

In conclusion, this study highlights the unmet needs within the RA treatment paradigm; patients may still experience lack of response to treatments, which incur HCRU and costs regardless of effectiveness. Of patients initiating their first biologic, 66% had an inadequate response by the end of their first year. These patients with inadequate response had numeric higher HCRU and non-medication direct healthcare costs. This analysis could provide information to key decision makers when developing reimbursement guidelines, which could help inform physicians’ decisions in clinical practice.

## References

[1] McInnes I, and Schett G. "The pathogenesis of rheumatoid arthritis." New England Journal of Medicine 365 23 (2011): 2205–2219. doi: 10.1056/NEJMra1004965 22150039 10.1056/NEJMra1004965

[2] Rudan I, Sidhu S, Papana A, Meng SJ, Xin-Wei Y, Wang W, et al Global Health Epidemiology Reference Group (GHERG). Prevalence of rheumatoid arthritis in low- and middle-income countries: A systematic review and analysis. J Glob Health. 2015 6;5(1):010409 doi: 10.7189/jogh.05.010409 25969732 10.7189/jogh.05.010409 PMC4416333

[3] Kuo CF, Luo SF, See LC, Chou IJ, Chang HC, Yu KH. Rheumatoid arthritis prevalence, incidence, and mortality rates: a nationwide population study in Taiwan. Rheumatol Int. 2013 2;33(2):355–60. doi: 10.1007/s00296-012-2411-7 22451027 10.1007/s00296-012-2411-7

[4] Lai CH, Lai MS, Lai KL, Chen HH, Chiu YM. Nationwide population-based epidemiologic study of rheumatoid arthritis in Taiwan. Clin Exp Rheumatol. 2012 May-Jun;30(3):358–63. 22513120

[5] Scott DL, Wolfe F, Huizinga TW. Rheumatoid arthritis. Lancet. 2010 9 25;376(9746):1094–108. doi: 10.1016/S0140-6736(10)60826-4 20870100 10.1016/S0140-6736(10)60826-4

[6] Hochberg M, Johnston S, John A. "The incidence and prevalence of extra-articular and systemic manifestations in a cohort of newly-diagnosed patients with rheumatoid arthritis between 1999 and 2006." Current medical research and opinion 24 2 (2008): 469–480. doi: 10.1185/030079908X261177 18179735 10.1185/030079908x261177

[7] Prete M, Racanelli V, Digiglio L, Vacca A, Dammacco F, Perosa F. Extra-articular manifestations of rheumatoid arthritis: An update. Autoimmun Rev. 2011 12;11(2):123–31. doi: 10.1016/j.autrev.2011.09.001 21939785 10.1016/j.autrev.2011.09.001

[8] Choy E, Kavanaugh A, Jones S. "The problem of choice: current biologic agents and future prospects in RA." Nature Reviews Rheumatology 9 3 (2013): 154–163. doi: 10.1038/nrrheum.2013.8 23419427 10.1038/nrrheum.2013.8

[9] Choy E. "Understanding the dynamics: pathways involved in the pathogenesis of rheumatoid arthritis." Rheumatology 51 suppl 5 (2012): v3–v11. 22718924 10.1093/rheumatology/kes113

[10] Dougados M, Soubrier M, Antunez A, Balint P, Balsa A, Buch MH, et al Prevalence of comorbidities in rheumatoid arthritis and evaluation of their monitoring: results of an international, cross-sectional study (COMORA). Ann Rheum Dis. 2014 1;73(1):62–8. doi: 10.1136/annrheumdis-2013-204223 24095940 10.1136/annrheumdis-2013-204223 PMC3888623

[11] Gabriel SE, Michaud K. Epidemiological studies in incidence, prevalence, mortality, and comorbidity of the rheumatic diseases. Arthritis Res Ther 2009;11:229 doi: 10.1186/ar2669 19519924 10.1186/ar2669 PMC2714099

[12] Gabriel SE. Why do people with rheumatoid arthritis still die prematurely? Ann Rheum Dis 2008;67:30–4. 10.1136/ard.2008.098038 PMC2830861 19022810

[13] Aviña-Zubieta JA, Choi HK, Sadatsafavi M, Etminan M, Esdaile JM, Lacaille D. Risk of cardiovascular mortality in patients with rheumatoid arthritis: a meta-analysis of observational studies. Arthritis Rheum. 2008 12 15;59(12):1690–7. doi: 10.1002/art.24092 19035419 10.1002/art.24092

[14] Lindhardsen J, Ahlehoff O, Gislason GH, Madsen OR, Olesen JB, Torp-Pedersen C, et al The risk of myocardial infarction in rheumatoid arthritis and diabetes mellitus: a Danish nationwide cohort study. Ann Rheum Dis. 2011 6;70(6):929–34. doi: 10.1136/ard.2010.143396 21389043 10.1136/ard.2010.143396

[15] Listing J, Gerhold K, Zink A. The risk of infections associated with rheumatoid arthritis, with its comorbidity and treatment. Rheumatology (Oxford) 2013;52:53–61. 23192911 10.1093/rheumatology/kes305

[16] Uresson C, Matteson EL. Malignancy as a comorbidity in rheumatic diseases. Rheumatology (Oxford) 2013;52:5–14. 22829694 10.1093/rheumatology/kes189

[17] Wotton CJ, Goldacre MJ. Risk of invasive pneumococcal disease in people admitted to hospital with selected immune-mediated diseases: record linkage cohort analyses. J Epidemiol Community Health 2012;66:1177–81. doi: 10.1136/jech-2011-200168 22493476 10.1136/jech-2011-200168

[18] Haugeberg G, Uhlig T, Falch JA, Halse JI, Kvien TK. Bone mineral density and frequency of osteoporosis in female patients with rheumatoid arthritis: results from 394 patients in the Oslo County Rheumatoid Arthritis register. Arthritis Rheum. 2000 3;43(3):522–30. doi: 10.1002/1529-0131(200003)43:3<522::AID-ANR7>3.0.CO;2-Y 10728744 10.1002/1529-0131(200003)43:3<522::AID-ANR7>3.0.CO;2-Y

[19] Coulson KA, Reed G, Gilliam BE, Kremer JM, Pepmueller PH. Factors influencing fracture risk, T score, and management of osteoporosis in patients with rheumatoid arthritis in the Consortium of Rheumatology Researchers of North America (CORRONA) registry. J Clin Rheumatol. 2009 6;15(4):155–60 doi: 10.1097/RHU.0b013e3181a5679d 19363452 10.1097/RHU.0b013e3181a5679d

[20] Lin YC, Li YH, Chang CH, Hu CC, Chen DW, Hsieh PH, et al Rheumatoid arthritis patients with hip fracture: a nationwide study. Osteoporos Int. 2015 2;26(2):811–7. doi: 10.1007/s00198-014-2968-y 25410437 10.1007/s00198-014-2968-y

[21] Bingham C, and Ruffing V. "Arthritis Information" Arthritis Information. Johns Hopkins, 24 9 2013 Web. 20 Sept. 2015. http://www.hopkinsarthritis.org/arthritis-info/rheumatoid-arthritis/ra-treatment/

[22] Smolen JS, van der Heijde D, Machold KP, Aletaha D, Landewé R. Proposal for a new nomenclature of disease-modifying antirheumatic drugs. Ann Rheum Dis. 2014 1;73(1):3–5. doi: 10.1136/annrheumdis-2013-204317 24072562 10.1136/annrheumdis-2013-204317

[23] Furst DE, and Emery P. "Rheumatoid arthritis pathophysiology: update on emerging cytokine and cytokine-associated cell targets." Rheumatology (2014): ket414. 10.1093/rheumatology/ket414 PMC4135582 24402580

[24] Strand V, Williams S, Miller PSJ, Saunders K, Grant S, Kremer JM. "OP0064 Discontinuation of Biologic Therapy in Rheumatoid Arthritis (RA): Analysis from the Consortium of Rheumatology Researchers of North America (CORRONA) Database." Annals of the Rheumatic Diseases 72 Suppl 3 (2013): A71–A72.

[25] Solomon G. "Implications for Rheumatoid Arthritis Treatment." Bulletin of the Hospital for Joint Diseases 71.3 (2013): 200–3. 24151945

[26] Emery P. "Optimizing outcomes in patients with rheumatoid arthritis and an inadequate response to anti-TNF treatment." Rheumatology 51 suppl 5 (2012): v22–v30. 22718923 10.1093/rheumatology/kes115

[27] Taiwan National Health Insurance Administration. Ministry of Health and Welfare. Immunologic Agents Reimbursement Guidelines. (2013). Available at: www.nhi.gov.tw/02hospital/hospital_file/chap8.doc

[28] Smolen JS, Breedveld FC, Burmester GR, Bykerk V, Dougados M, Emery P, et al Treating rheumatoid arthritis to target: 2014 update of the recommendations of an international task force. Ann Rheum Dis. 2016 1;75(1):3–15. doi: 10.1136/annrheumdis-2015-207524 25969430 10.1136/annrheumdis-2015-207524 PMC4717393

[29] Lai NS, Tsai TY, Li CY, Koo M, Yu CL, Lu MC. Increased frequency and costs of ambulatory medical care utilization prior to the diagnosis of rheumatoid arthritis: a national population-based study. Arthritis Care Res (Hoboken). 2014 3;66(3):371–8. 23983071 10.1002/acr.22146

[30] Chang JY, Lang HC. "The Econimic Cost of Rheumatoid Arthritis in Taiwan." Value in Health 17 7 (2014): A773. 10.1016/j.jval.2014.08.328 27202853

[31] Wang BC, Hsu PN, Furnback W, Ney J, Yang YW, Fang CH, et al Estimating the Economic Burden of Rheumatoid Arthritis in Taiwan Using the National Health Insurance Database. Drugs Real World Outcomes. 2016;3:107–114. 10.1007/s40801-016-0063-8 PMC4819475 27747810

[32] Curtis JR, Baddley JW, Yang S, Patkar N, Chen L, Delzell E, et al Derivation and preliminary validation of an administrative claims-based algorithm for the effectiveness of medications for rheumatoid arthritis. Arthritis Res Ther. 2011;13(5):R155 doi: 10.1186/ar3471 21933396 10.1186/ar3471 PMC3308085

[33] Deyo RA, Cherkin DC, Ciol MA. Adapting a clinical comorbidity index for use with ICD-9-CM administrative databases. J ClinEpidemiol. 1992 6;45(6):613–9. 10.1016/0895-4356(92)90133-8 1607900

[34] New Taiwan Dollar (NT$) → US Dollar ($). Google Finance. Accessed May 30, 2017. Available at: < https://www.google.com/finance?q=TWDUSD&ei=WJdIWbDLNYjGmAHLvZ-oCA >

[35] Moots RJ, Xavier RM, Mok CC, Rahman MU, Tsai WC, Al-Maini MH, et al The impact of anti-drug antibodies on drug concentrations and clinical outcomes in rheumatoid arthritis patients treated with adalimumab, etanercept, or infliximab: Results from a multinational, real-world clinical practice, non-interventional study. PLoS One. 2017 4 27;12(4):e0175207 eCollection 2017. Erratum in: PLoS One. 2017 Jun 5;12 (6):e0179308. doi: 10.1371/journal.pone.0175207 28448562 10.1371/journal.pone.0175207 PMC5407581

[36] Hsieh CR, Liu YM. "Availability, Health-Care Costs, and Utilization Patterns of Biologics in Taiwan." Value in Health 15 1 (2012): S35–S42. 22265065 10.1016/j.jval.2011.11.025

